# Modulating scar formation for improving brain repair: from coagulation and inflammation to cell therapy

**DOI:** 10.1007/s00441-022-03601-4

**Published:** 2022-02-28

**Authors:** Christian Schachtrup

**Affiliations:** 1 Department of Molecular Embryology, Institute of Anatomy and Cell Biology, University of Freiburg, Albertstrasse 17, 79104 Freiburg, Germany; 2 Center for Basics in NeuroModulation (NeuroModulBasics), Faculty of Medicine, University of Freiburg, Freiburg, Germany

## Introduction

During the past decade, the field has made impressive progress in understanding the multicellular organization and interactions among multiple types of CNS glia, non-neural stromal cells, and the vasculature by especially next-generation sequencing technology and functional studies, shining light on innovations for manipulating the scar formation and beneficial therapeutic approaches. The wound-healing process in the CNS typically starts with an inflammatory response by tissue-resident cells. A coagulation cascade is activated to achieve hemostasis, leukocytes infiltrate to clear debris, and glial cells proliferate to form a barrier to separate functioning neural tissue from meningeal stromal cells and restrain the propagation of inflammation. The different stromal cells of the perivascular niche generate pathological fibroblasts that deposit excessive fibrotic extracellular matrix (ECM) that hinders tissue remodeling and functional recovery.

As we strive to understand the biological complexity of scar tissue formation and tissue fibrosis in the CNS, we clearly cannot study glia, non-neural stromal cells, and extracellular matrix in isolation. Current therapies address some aspects of scar formation to improve repair, but none encourages regeneration. One of the main lines of inquiry with the regenerative medicine is how to best leverage neural stem cells (NSCs) and tissue engineering scaffold material as potential therapy for scar formation in human neurological conditions. We know, for example, little about the molecular mechanisms of endogenous or transplanted NSC survival and integration and their cell fate determination and functionality towards the modification and resolution of the glial and fibrotic scar. Yet, understanding these aspects is for harnessing these cells for brain repair.

This Special Issue highlights several of the recent advances in understanding scar formation and regeneration. The complex picture of molecular mechanisms of the genesis of glial and fibrotic scarring is now better understood. Novel NSC mechanisms of action to modify the pro-inflammatory environment have been identified. New insights into the roles of engineering strategies towards overcoming bleeding and scar formation as well as optimized combinations of NSC-biomaterial-based scaffolds have been achieved, suggesting that NSC transplantation could be beneficial in the resolution and regeneration of the scar. The purpose of this collection of reviews is not to present a comprehensive overview of the current status of knowledge on scar formation. Rather, it is the guest editors’ concept to focus on recent progress seen from the perspective of studying scar formation through the multidisciplinary prism of vascular biology, neuroscience, stem cell biology, and engineering strategies, which might be for identifying novel mechanisms of disease pathogenesis, developing new animal models, detecting biomarkers, and discovering therapeutic treatments.

## A brief overview of articles in this special issue

The sheer number of cell types involved in the formation of the glial/fibrotic scar highlights the many hurdles required for post-spinal cord injury regeneration and ultimate functional recovery. In the first part of the review, Tran et al. (2021) summarize how the capacity of CNS regeneration changes across phyla and ages through formation of scar-like structures. Then, the authors explore how the new insights from next-generation sequencing technology, along with experimental manipulations, have yielded a more complex portrait of the molecular mechanisms governing the responses of astrocytes, microglia, and neurons to injury and development, especially of the glial component of the scar. Finally, they explore how chondroitin sulfate proteoglycans impede regeneration/plasticity and how combinatorial therapeutic approaches centering on scar modulation might restore function after severe CNS injury.

Clearly, CNS responses to insults involve complex interactions among diverse neural and non-neural cells. Wahane and Sofroniew (2021) discuss the effects on CNS scar formation and tissue repair in loss-of-function studies with specific cell types in in vivo models of disorder. Various CNS glial cell types are essential to repair CNS tissue. They are interacting to surround and corral stromal cell scars and fibrosis, and manipulations that hinder their functions yield detrimental effects. The authors then highlight similarities among CNS scars, limitans borders, and secondary lymphoid follicles, which might instruct us to further dissect mechanisms of CNS scar formation. The authors note that CNS glia, as well as fibrosis-producing stromal cells, are emerging as major contributors to diverse CNS disorders by either loss- or gain-of-functions and are, therefore, important potential targets for interventions.

Recent studies demonstrating the perivascular niche as a source of fibroblasts have debunked the traditional view that a fibrotic scar only forms after penetrating lesions with torn meninges. Fehlberg and Lee (2021) describe the perivascular cell origin contributing to the fibrotic scar and review the development of fibrosis across a spectrum of CNS injuries. The molecular mechanisms underlying CNS fibrosis seem to share many features with fibrosis in other organs: the process is mediated by an inflammatory response, whereas IFNγ and TGF-β secreted by inflammatory cells seem to be the major drivers that activate fibroblasts. Understanding fibrosis in other organs should provide important insights into how CNS fibrosis can be therapeutically targeted to promote functional recovery. However, like astrogliosis, CNS fibrosis has neuroprotective effects, and therefore, understanding CNS fibrosis in the larger context of wound healing is necessary to develop appropriate treatment strategies.

As with other fibrotic diseases, subretinal fibrosis in neovascular age-related macular degeneration (nAMD) is a consequence of chronic tissue injury. Tenbrock et al. (2021) review current concepts, therapeutic avenues, and future perspectives on subretinal fibrosis in nAMD, which is a progressive, degenerative disease leading in its most aggressive form to irreversible blindness. They describe the clinical manifestations of nAMD and the current understanding of the underlying cellular and molecular mechanisms. They emphasize the contribution of myofibroblasts and the potential source of myofibroblast precursors, as well as the immune response and growth factor contributions to subretinal fibrosis. Although no anti-fibrotic treatment reduces the subretinal scarring in patients with nAMD, early detection and combinatorial therapies, such as anti-PDGF and anti-VEGF combination therapy, offer significant hope for inhibiting subretinal fibrosis in the future.

Microglia act as a line of defense in the CNS by phagocytizing harmful pathogens and cellular debris and by releasing a variety of factors that mediate immune responses. They are also critical mediators of neurophysiology, making them a desired target to rectify neuropathological states. Neckles and Feliciano (2021) discuss microglia ontogenesis with a particular focus on microglia of the subventricular zone (SVZ) stem cell niche and use this knowledge for targeting strategies of microglia developmental pathways for brain repair. The authors review how extracellular factors (EVs) regulate microglia development and, more specifically, how EVs secreted by astrocytes and NSCs alter microglia physiology during development. The interactions of these two types of EVs with microglia affecting microglia physiology might be harnessed in the future to recruit or remove microglia from neuroanatomical sites to rectify neuropathological states.

Endothelial cells (ECs) act as a dynamic lining to control the complex interplay of the coagulation system with the surrounding cells, including immune cells. Neubauer and Zieger (2021) focus on specific functions and adaptations of ECs that maintain blood fluidity and control vascular hemostasis at sites of injury. When the vasculature is damaged, the endothelium shifts from an anticoagulant to a procoagulant/prothrombotic phenotype to prevent excessive blood loss. Hemostasis begins almost instantly, thus stopping blood loss and allowing repair to begin. The authors describe the two processes involved in hemostasis, platelet activation and the coagulation system, in detail. ECs facilitate multiple mechanisms, including procoagulant and anticoagulant, by actively regulating the blood coagulation system and the production of solutes, hormones, or macromolecules.

NSCs hold great promise for improving CNS repair, by either triggering endogenous NSC sources of the CNS or transplanting exogenous NSCs. The molecular mechanisms of NSC cell-fate determination and their cross-communication with glial and fibrotic scar cells are still insufficiently understood, but these will be instrumental for harnessing these cells as regenerative therapies in glial scar and brain repair. Nicaise et al. (2021) review the pathobiology of the glial scar, the cellular components and extracellular matrix of the glial scar, and current findings on endogenous and exogenous NSCs in glial scar biology. They emphasize that, beside its trophic support, NSC transplantation could be beneficial in the resolution of the glial scar, possibly by targeting the metabolic machinery of myeloid cells. Towards the challenge in studying exogenous transplantation of NSCs in understanding how this technology can be realistically be brought to clinic, the authors first summarize human cell sources for NSC transplantation and finally introduce novel 3D stem cell–based technologies to model this pathology in a dish.

Mechanisms that direct the migration of endogenous NSCs towards a lesion area are poorly understood. In their paper, Deshpande et al. (2021) suggest that the p75 neurotrophin receptor (p75^NTR^) controls the migration of NSCs from the SVZ to the lesion site by regulating the cytoskeleton after cortical injury. Genetic depletion of p75^NTR^ in mice reduced that migration, and p75^NTR^-deficient NSCs failed to migrate upon BDNF stimulation in vitro. Cortical injury rapidly increased p75^NTR^ abundance in SVZ NSCs via bone morphogenetic protein receptor signaling. SVZ-derived p75^NTR^-deficient NSCs revealed an altered cytoskeletal network and an altered expression of genes and proteins of the small GTPase family. In agreement with that, BMP-treated non-migrating p75^NTR^-deficient NSCs revealed an altered morphology and α-tubulin expression, compared to BMP-treated migrating wild-type NSCs. Thus, BMP-induced p75^NTR^ abundance in the NSCs may be a sensor for increased ligands in cortical lesions and may provide a therapeutic target for directed migration of NSCs and improving brain repair.

Environmental changes after CNS injury alter NSC cell fate and interfere with their pro-repair functions. In their review, Chu et al. (2021) describe the transcriptional control of adult SVZ NSC differentiation in CNS disease. More specifically, they focus on the inhibitor of DNA binding (Id) proteins, which act by antagonizing the DNA-binding activity of basic helix-loop-helix transcription factors to determine cell-fate decisions in NSCs. Id protein expression is mainly triggered and stabilized by the TGF-β superfamily and regulates NSC and oligodendrocyte progenitor cell fate in CNS injury and disease. Although Id proteins were formerly considered undruggable, recent developments in small-molecule compounds regulating Id-E protein interactions allow control of Id levels in glial cells and NSCs. Thus, pharmacological intervention involving Id proteins has the potential to modify stem cell behavior and ameliorate CNS disease.

Engineering strategies for modifying scar formation include optimized combinations of biomaterial-based scaffolds. Fibrin is a natural matrix that provides the first structure for the initiation of repairs after injury. Not surprisingly, it provides attractive biological conditions for the therapeutic delivery of progenitor cells and morphogens in many fields of regenerative medicine. Melly and Banfi (2022) provide an overview of the use of fibrin as a tissue-engineering scaffold and a tunable platform for the fine control of dose and duration of delivery of recombinant factors in therapeutic angiogenesis. This review introduces therapeutic angiogenesis, describes fundamental biological concepts in angiogenesis, and summarizes the therapeutic requirements for angiogenic factor delivery. While fibrin hydrogels for vascularization in regenerative medicine embody several useful features, they are also associated with undesirable outcomes: under some conditions, fibrin activates anti-regenerative processes and stimulates fibrosis and scar formation. Understanding the mechanisms that tip the balance between the pro- and anti-regenerative functions of fibrin will be key to fully exploit its therapeutic potential.

Neural probes are sophisticated electrophysiological tools used for intra-cortical recording and stimulation. One of the challenges and currently most significant limitations is their “seamless” long-term integration into the surrounding tissue. Otte et al. (2022) in their review emphasize that, after implantation, which is typically accompanied by bleeding, the tissue responds with a scarring process. They summarize the considerable engineering progress that has minimized this reaction. These include improved materials, microfabrication, and surgical techniques, novel, tailored probes, and a better understanding of the immune response to their implantation. However, the question of whether bleeding and scar formation are inevitable remains open. Optimal surgical methods with flexible probes and/or minimized cross section of the single shanks ameliorate glial scar formation, and these improvements might be sufficient for long-term, stable, functional interfacing of neurons even when the implant is not entirely scar-free.

The neurovascular interface transforms dramatically into a pro-inflammatory niche to trigger the coagulation cascade and establish hemostasis after CNS injury. These actions might initiate glial scar formation and tissue fibrosis in the CNS. Fibrinogen, the coagulation factor I, serves as the major architectural protein component of blood clots and, in addition, promotes inflammation and neurodegeneration. Galanakis et al. (2022) characterize soluble fibrin (SF), which in blood consists of monomers lacking both fibrinopeptides A with a minor population in multimeric clusters. SF is a substantial component of isolated fibrinogen that is elevated in inflammation and a risk of thrombotic complications. They show that spontaneously generated SF fibers form intercellular links culminating in rouleaux formation and erythrocyte sedimentation acceleration, which reflects hypercoagulability in clinical settings. Their results explain fibrinogen binding to erythrocytes via CD47, stable in vivo erythrocyte aggregates in capillaries and red areas of pathologic thrombi. A detailed understanding of fibrin and fibrinogen structure, cellular targets, and signaling networks is the prerequisite for selective drug targeting to suppress the damaging functions of fibrinogen in the CNS without affecting the beneficial effects in hemostasis.

The relevance of the coagulation cascade and coagulation factors was not considered for a long time in neuroscience, at least with regard to their role in defining physiological brain functions, such as neural excitability and synaptic plasticity. Maoz et al. (2021) review how “brain born” coagulation factors affect brain physiology and pathology. They focus on the role of thrombin/PAR1 in synaptic plasticity and how its misregulation in changes to the blood–brain interface affects complex brain functions. They further describe the major limitations of current experimental and clinical models to study the cellular and molecular mechanisms of the brain-born coagulation factors in complex neurovascular unit functions. Instead, they propose to study these mechanisms on an organ-on-a-chip platform. The authors hope to capitalize on the rapid progress within neurotechnology to advance the electrophysiology and biosensing function of organ-on-a-chip platforms that might allow them to identify and probe complex cell–cell interactions in the neurovascular unit.

In summary, in this Special Issue, leading researchers describe the latest findings on glial/fibrotic scar formation, the challenges of multicellular and complex molecular interactions in the scar to promising stem cell–based therapies, and insights into the role of blood and coagulation factors as critical players in CNS injury and repair. A comprehensive understanding of the cellular interplay at the pro-inflammatory niche and after NSC/biomaterial or probe transplantation during scar formation will facilitate novel therapeutic approaches for better regeneration and, in addition, facilitate cutting-edge developments for tissue engineering and implantation technology. Finally, the shared hope is that this knowledge will benefit and stimulate the field to elaborate new ideas that advance in the future.

## References

[CR1] Chu YH, Lin JD, Nath S, Schachtrup C (2021). Id proteins: emerging roles in CNS disease and targets for modifying neural stemcell behavior. Cell Tissue Res.

[CR2] Deshpande SS, Malik SC, Conforti P, Lin JD, Chu YH, Nath S, Greulich F, Dumbach MA, Uhlenhaut NH, Schachtrup C (2021). P75 neurotrophin receptor controls subventricular zone neural stem cell migration after stroke. Cell Tissue Res.

[CR3] Fehlberg CR, Lee JK (2021). Fibrosis in the central nervous system: from the meninges to the vasculature. Cell Tissue Res.

[CR4] Galanakis DK, Protopopova A, Li K, Yu Y, Ahmed T, Sensel L, Heslin R, Gouda M, Koo J, Weisel J, Manco-Johnson M, Rafailovich M (2022). Novel characteristics of soluble fibrin: hypercoagulability and acceleration of blood sedimentation rate mediated by its generation of erythrocyte-linked fibers. Cell Tissue Res.

[CR5] Maoz BM, Asplund M, Maggio N, Vlachos A (2021). Technology-based approaches toward a better understanding of neuro-coagulation in brain homeostasis. Cell Tissue Res.

[CR6] Melly L, Banfi A (2022). Fibrin-based factor delivery for therapeutic angiogenesis: friend or foe?. Cell Tissue Res.

[CR7] Neckles VN, Feliciano DM (2021). From seed to flower: blossoming of microglia in development and brain repair. Cell Tissue Res.

[CR8] Neubauer K, Zieger B (2021). Endothelial cells and coagulation. Cell Tissue Res.

[CR9] Nicaise AM, D'Angelo A, Ionescu RB, Krzak G, Willis CM, Pluchino S (2021). The role of neural stem cells in regulating glial scar formation and repair. Cell Tissue Res.

[CR10] Otte E, Vlachos A, Asplund M (2022). Engineering strategies towards overcoming bleeding and glial scar formation around neural probes. Cell Tissue Res.

[CR11] Tenbrock L, Wolf J, Boneva S, Schlecht A, Agostini H, Wieghofer P, Schlunck G, Lange C (2021). Subretinal fibrosis in neovascular age-related macular degeneration: current concepts, therapeutic avenues, and future perspectives. Cell Tissue Res.

[CR12] Tran AP, Warren PM, Silver J (2021). New insights into glial scar formation after spinal cord injury. Cell Tissue Res.

[CR13] Wahane S, Sofroniew MV (2021) Review article for CTR special issue edited by C. Schachtrup Title of Special Issue: “Modulating scar formation for improving brain repair” loss-of-function manipulations to identify roles of diverse glia and stromal cells during CNS scar formation. Cell Tissue Res. 10.1007/s00441-021-03487-8 10.1007/s00441-021-03487-8 PMC8975763 34164732

